# Physicochemical Properties and Biological Activities of Polysaccharides from *Dendrobium officinale* Leaves in Response to Different Extraction Methods

**DOI:** 10.3390/foods14122029

**Published:** 2025-06-08

**Authors:** Yang Chen, Gonglin Cai, Hufu Chen, Huabin Zhou, Hang Qu, Hailong Yang

**Affiliations:** 1 College of Life & Environmental Science, Wenzhou University, Wenzhou 325035, China; 2 Zhejiang XiangHai Food Co., Ltd., Wenzhou 325000, China; 3 Zhejiang Provincial Key Laboratory of Water Environment Ecological Governance and Resource Protection, Wenzhou University, Wenzhou 325035, China

**Keywords:** *Dendrobium officinale* leaves, polysaccharide, extraction method, physicochemical properties, antioxidant activity, immunostimulatory activity

## Abstract

Extraction techniques play a crucial role in determining the structural attributes and biological functions of polysaccharides. This study aimed to evaluate the physicochemical and biological properties of *Dendrobium officinale* leaf polysaccharides (DLPs) extracted using various methods, including hot water, acidic, alkali, ultrasound-assisted, and enzyme-assisted extraction. The results indicated that the extraction methods significantly influenced the yield, content, molar ratios of monosaccharides, molecular weight, and structural features of the polysaccharides, as well as their in vitro adsorption, antioxidant, and immunostimulatory activities. Among these extraction methods assessed, enzyme-assisted extraction yielded the highest amount of polysaccharides, characterized by a substantial galacturonic acid residue and exceptional scavenging capability towards free radicals. In contrast, hot water extraction and ultrasound-assisted extraction preserved a triple helix conformation, enabling the polysaccharides to exhibit superior adsorption capabilities for cholesterol and nitrite, as well as significantly increasing the release of nitric oxide and the secretion of TNF-α, IL-6, and IL-1β in RAW264.7 macrophages. These findings suggest that enzyme-assisted, ultrasound-assisted, and hot water extraction are effective approaches to extract DLPs with pronounced biological activity. The selection of the extraction method for DLPs should be tailored to align with the specific requirements of practical applications.

## 1. Introduction

*Dendrobium officinale* (*D. officinale*) is a perennial epiphytic herb classified within the genus *Dendrobium* of the Orchidaceae family. This species is primarily distributed in tropical and subtropical regions, including China, India, and Malaysia [1]. As a valuable medicinal and edible plant, *D. officinale* is widely utilized in traditional Chinese medicine and functional foods due to its rich bioactive compounds, such as polysaccharides, polyphenols, and alkaloids [2]. The low reproductive rate of *D. officinale* under natural conditions fails to meet the increasing market demand. Consequently, large-scale cultivation has been gradually developed in recent years to ensure the sustainable use of *D. officinale* resources. Currently, the cultivation area of *D. officinale* spans approximately 13,000 ha in China, generating an annual output value of around 200 million US dollars [3]. Notably, the *D. officinale* industry primarily focuses on its flowers and stems, while the leaves, which constitute about 50% of the plant’s biomass, are often overlooked and discarded [4]. However, a growing body of research has demonstrated that the polysaccharides derived from *D. officinale* leaves possess significant biological activities, including antioxidant, antitumor, hypolipidemic, and immunomodulatory effects [5,6].

Polysaccharides are natural complex polymers, and their physicochemical characteristics and biological activities are closely linked to the methods used for their extraction [1,7]. The methods for plant polysaccharide extraction can be classified based on the extracting solvents (e.g., water, acid, alkali, supercritical fluid) [8,9], physical assistance (e.g., ultrasonic, heating, microwave) [10], biological assistance (e.g., enzyme-assisted extraction) [11], or composite methods that combine these techniques. In the polysaccharide extraction process, key factors such as solvent penetration, diffusion rate, and the disruption of the cell wall play crucial roles. Among these, the solvent’s penetration capacity and the diffusion rate of the solute are particularly decisive [12]. Hot water extraction is the most commonly used method due to its simplicity and low equipment requirements [13]. Other methods, such as acid extraction, alkaline extraction, ultrasound-assisted extraction, and enzyme-assisted extraction, are extensively employed due to their ability to disrupt the plant cell wall, facilitate the diffusion of polysaccharides into the solvent, and modify the polysaccharide structure, potentially enhancing their biological activity [14]. Despite the growing interest in *D. officinale* leaf polysaccharide (DLP), most studies have focused on a single extraction method, such as hot water extraction, with limited comparisons of the physicochemical properties and biological activities of polysaccharides extracted using different methods [15,16]. Therefore, conducting a comparative study of various extraction techniques is essential for understanding their impacts on the characteristics of DLP.

In this study, five distinct extraction methods—hot water extraction, ultrasound-assisted extraction, enzyme-assisted extraction, acid extraction, and alkaline extraction—were employed to obtain DLP extracts. A comprehensive analysis and comparison of the physicochemical properties and biological efficacy of the polysaccharide extracts obtained through these various methods were conducted. The results of this research will provide a theoretical foundation for the preparation of DLP with enhanced biological activity.

## 2. Materials and Methods

### 2.1. Materials

*D. officinale* leaves were collected from the Yueqing Greenfeng *D. officinale* Cultivation Cooperative (Wenzhou, China) in May 2024. The leaves were hot-air dried at 50 °C, then pulverized and sieved through a 40-mesh sieve.

### 2.2. Extraction of Polysaccharides

**Figure 1:**
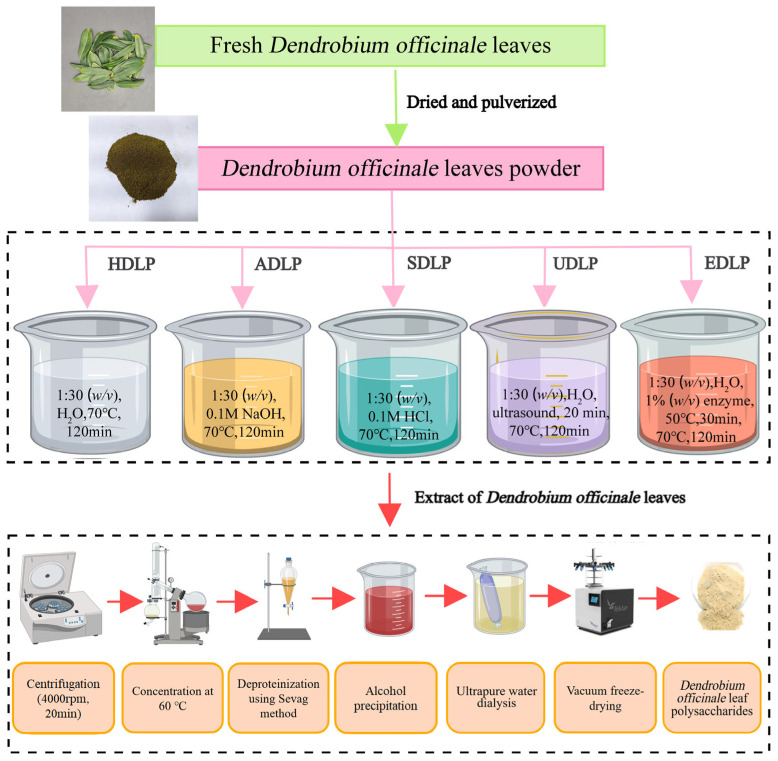
Flowchart for the preparation of *Dendrobium officinale* leaf polysaccharides extracted using different methods.

*D. officinale* leaf polysaccharides (DLPs) were extracted using different methods, including hot water extraction, ultrasound-assisted extraction, enzyme-assisted extraction, acid extraction, and alkaline extraction. A flowchart illustrating the preparation process is presented in Figure 1.

#### 2.2.1. Hot Water Extraction

Dried powder of *D. officinale* leaves was added to deionized water at a ratio of 1:30 (*w*/*v*). The hot water extraction was conducted under atmospheric pressure at 70 °C for 2 h, with stirring at 500 rpm. The supernatant was obtained through centrifugation using a centrifuge (Anting Scientific Instrument Factory, Shanghai, China) at 4000 rpm for 20 min. This extraction process was repeated, and the resulting supernatants were combined and concentrated using a rotary evaporator (Yarong Biochemical Instrument Factory, Shanghai, China) at 60 °C. Proteins in the concentrate were removed using the Sevag method (chloroform: butanol = 1:4) three times. The aqueous phase was collected, and four volumes of anhydrous ethanol were added. After standing at 4 °C overnight, the precipitate was collected by centrifugation, dialyzed in a 3500 Da dialysis bag (Solarbio Science Co., Ltd., Beijing, China) for 48 h at 4 °C, and then dried using a freeze dryer to obtain hot water extracted *D. officinale* leaf polysaccharide (HDLP) [5].

#### 2.2.2. Ultrasound-Assisted Extraction

The ultrasound-assisted extraction was performed according to the method described by Fu et al. [17], with minor modifications. Briefly, *D. officinale* leaves powder was mixed with deionized water (1:30, *w*/*v*), and subjected to ultrasonic extraction at a temperature of 70 °C and a power of 450 W for 20 min using an ultrasonic machine (Kunshan Ultrasonic Instrument Co., Ltd., Suzhou, China). Subsequently, the procedure was carried out as described in Section 2.2.1 to obtain the ultrasound-assisted extracted polysaccharide (UDLP).

#### 2.2.3. Acid and Alkali Extraction

The dried powder of *D. officinale* leaves was added to 0.1 mol/L hydrochloric acid (HCl) solution (pH 1.8) and 0.1 mol/L sodium hydroxide (NaOH) solution (pH 12.5) at a ratio of 1:30 (*w*/*v*) [18]. The extraction process was carried out as detailed in Section 2.2.1 to obtain the acid extracted polysaccharide (SDLP) and alkali extracted polysaccharide (ADLP).

#### 2.2.4. Enzyme-Assisted Extraction

For cellulase-assisted extraction, dried *D. officinale* leaves powder was combined with deionized water at a ratio of 1:20 (*w*/*v*). The pH was adjusted to 5.0 using 0.1 mol/L HCl and NaOH solutions, after which 1% cellulase (Pangbo Bioengineering, Nanning, China) was added. The mixture was incubated at 50 °C for 30 min, followed by heating to 95 °C for 5 min to inactivate the enzyme [19]. Subsequently, the material-to-liquid ratio was adjusted to 1:30 (*w*/*v*), and the extraction was performed as described in Section 2.2.1 to prepare the enzyme-assisted extracted *D. officinale* leaf polysaccharide (EDLP).

### 2.3. Physicochemical Characteristics Analyses

#### 2.3.1. Chemical Composition

The lyophilized polysaccharides obtained from different extraction methods were dissolved in deionized water at a concentration of 2 mg/mL. The polysaccharide content was quantified using the phenol-sulfuric acid method [9]. The standard curve was y = 0.6095x + 0.0025 (R^2^ = 0.9952). The extraction yield was calculated using the equation: yield(%) = (C × V × D)/m, where C denotes the concentration of DLP, V denotes the volume of DLP solution, and m denotes the initial dry weight of *D. officinale* leaves. The glucuronic acid content was analyzed using the *m*-hydroxybiphenyl method, with D-glucuronic acid as the reference standard [20]. The protein content was determined using a BCA assay kit (Beyotime Biotechnology, Shanghai, China) [11].

#### 2.3.2. Molecular Weight Determination

The molecular weight was analyzed using a 1260 HPLC system (Agilent Technologies, Santa Clara, CA, USA) equipped with a TSK gel G400PWXL column (10 µm, 7.8 × 300 mm, Tokyo, Japan) and a differential refractive index (RI) detector. Standard dextran solutions of varying molecular weights and DLPs were prepared at a concentration of 5 mg/mL and then filtered through a 0.45 μm microporous filter membrane for analysis. An injection volume of 20 µL was utilized. The mobile phase was ultrapure water at a flow rate of 0.5 mL/min. The column temperature was set to 30 °C. The molecular weight of the polysaccharide was calculated based on the regression equation derived from the standard curve.

#### 2.3.3. Monosaccharide Composition Analysis

The monosaccharide composition was analyzed as previously reported [21]. Briefly, the polysaccharide was hydrolyzed using 4 mol/L of trifluoroacetate (TFA) at 110 °C for 4 h, labeled with 1-phenyl-3-methyl-5 pyrazolone (PMP), and analyzed using a 1260 HPLC system equipped with a Hypersil ODS column (250 × 4.6 mm, 5 μm) and a diode array detector (DAD). The mobile phase consisted of a 0.1 mol/L phosphate buffer (pH 6.8) and acetonitrile in at ratio of 83:17 (*v*/*v*), with a flow rate of 1.0 mL/min. The injection volume was 10 μL, and the column temperature was maintained at 30 °C. The detection wavelength was set at 250 nm.

#### 2.3.4. Congo Red Test

To identify the triple helical structure of the polysaccharide, a Congo red test was conducted following the method described by Geng et al. with slight modifications [22]. Briefly, 1 mL of polysaccharide solution (2 mg/mL) and 1 mL of Congo red (Sinopharm Chemical Reagent, Shanghai, China) solution (0.1 mmol/L) were mixed with varying volumes (0, 0.4, 0.8, 1.2, 1.6, and 2.0 mL) of NaOH solution. After reaction for 30 min at room temperature, the maximum absorption wavelength of the mixture was recorded by spectral scanning in the range of 400 nm to 700 nm using a spectrophotometer (Purkinje General Instrument, Beijing, China).

#### 2.3.5. Scanning Electron Microscopy (SEM)

The polysaccharide sample was coated with a thin layer of gold under reduced pressure and subsequently photographed using a SEM (NanoSEM200, Field Electron and Ion Company, FEI, Hillsboro, OR, USA) at an accelerating voltage of 10 kV with appropriate magnification [23].

#### 2.3.6. Fourier Infrared (FT-IR) Spectral Scanning

An amount of polysaccharide was mixed with KBr powder, thoroughly milled, pressed into tablets, and analyzed using a TENSOR FT-IR spectrometer (Bruker, Ettligen, Germany) over the range of 400 cm^−1^ to 4000 cm^−1^ at a resolution of 4.0 cm^−1^, with a background scanning frequency of 64 [24].

### 2.4. Adsorption Capacity Determination

#### 2.4.1. Cholesterol Adsorption Capacity (CAC)

The cholesterol adsorption capacity (CAC) of DLPs was measured using a previously established method [25]. In brief, fresh egg yolk was mixed with distilled water in a ratio of 1:9 (*w*/*v*) and homogenized to create an emulsion solution. The pH of the solution was then adjusted to 2.0 and 7.0 to simulate the pH conditions of gastric juice and the intestinal tract, respectively. These resulting solutions served as blank solutions before adsorption. Thereafter, 0.1 g of polysaccharide was added to the blank solutions, and the mixtures were incubated in a shaker at 120 rpm and 37 °C for 2 h. The residual cholesterol was quantified using the *o*-phthalaldehyde method at 550 nm after removing the polysaccharide by ethanol precipitation. The CAC was calculated using the equation: CAC (mg/g) = (M_1_ − M_2_)/M, where M_1_ and M_2_ represent the amounts of cholesterol before and after absorption in the solution, respectively, and M denotes the weight of the polysaccharide.

#### 2.4.2. Nitrite Adsorption Capacity (NAC)

The nitrite adsorption capacity (NAC) of DLPs was measured according to the method described by Gan et al. [26]. Briefly, 0.1 g of polysaccharides was dissolved in 5.0 mL of sodium nitrite solution (20 µg/mL), and the pH was adjusted to 2.0 and 7.0 to simulate the pH levels of gastric juice and the intestinal tract, respectively. The resulting solutions served as blank solutions before adsorption. After shaking for 2 h at 120 rpm and 37 °C, the polysaccharides were precipitated with ethanol. The residual nitrite was measured using 400 µg/mL of *p*-aminobenzene sulfonic acid (Xilong Chemical, Shanghai, China) and 200 µg/mL of hydrochloride naphthodiamide (Sinopharm Chemical Reagent, Shanghai, China) reagents at a wavelength of 538 nm. The NAC was calculated as follows: NAC (µg/g) = (M_1_ − M_2_)/M, where M_1_ and M_2_ represent the amounts of nitrite before and after absorption in the solution, respectively, and M denotes the weight of the polysaccharide [25].

### 2.5. Antioxidant Activity Analysis

The antioxidant activity of polysaccharide was evaluated by examining the scavenging ability towards 1,1-diphenyl-2-picrylhydrazyl (DPPH), 2,2′-azinobis (3-ethylbenzothiazoline- 6-sulfonic acid) (ABTS) and hydroxyl radicals. The scavenging activities towards DPPH and ABTS radicals were determined in accordance with the method of Cai et al. [27], while the hydroxyl radical scavenging capacity was assessed using the method outlined by Geng et al. [22]. Vitamin C (Xilong Chemical, Shanghai, China) served as a positive control. The results were expressed as the IC_50_ value, indicating the concentration of the sample required to achieve 50% inhibition of free radical activity.

### 2.6. Immunostimulatory Activity Analysis

#### 2.6.1. Cell Culture

RAW 264.7 macrophages were purchased from Pricella Life Science and Technology Co., Ltd. (Wuhan, China). These cells were cultured in Dulbecco’s modified Eagle’s medium (DMEM) (Thermo Fisher Scientific, Waltham, MA, USA) supplemented with 10% fetal bovine serum (FBS) and 1% penicillin/streptomycin, and maintained at 37 °C in an atmosphere containing 5% CO_2_.

#### 2.6.2. Cell Viability Assay

RAW264.7 cells were seeded in 96-well plates at a density of 8000 cells per well and cultured until they adhered to the plate surface. The cells were treated with polysaccharides at concentrations of 50, 100, 200, 400, 600, and 800 µg/mL, or with lipopolysaccharide (LPS) at a concentration of 1 μg/mL (Solarbio Life Sciences, Beijing, China) for 24 h. LPS served as a positive control. Subsequently, the cytotoxicity of the polysaccharides on the cells was determined using the CCK-8 assay (Beyotime Biotechnology, Shanghai, China) [28]. Cell viability was calculated using the following equation: cell viability (%) = (A_1_/A_0_) × 100%, where A_0_ and A_1_ represent the absorbance values of the negative control group and the test group, respectively.

#### 2.6.3. Phagocytosis

The effect of polysaccharide on the phagocytosis of RAW 264.7 cells was assessed using the method of neutral red [29]. Briefly, the cells were treated with 200 μg/ mL of polysaccharide solution or 1 μg/ mL of LPS for 24 h, after which the culture medium was discarded. Thereafter, a 0.075% neutral red (Solarbio Life Sciences, Beijing, China) solution was introduced and allowed to incubate for 1 h. The cells were then rinsed twice with phosphate buffered saline (PBS) and treated with lysis buffer (acetic acid: ethanol = 1:1, *v*/*v*). The absorbance was measured at 540 nm.

#### 2.6.4. NO, TNF-α, IL-1β and IL-6 Concentrations Determination

RAW 264.7 cells were subjected to treatment with polysaccharides extracted using different methods at a concentration of 200 µg/mL. Following a 24 h-incubation, the cell supernatants were collected for analysis. The levels of nitric oxide (NO) were quantified using a NO assay kit (Beyotime Biotechnology, Shanghai, China). Additionally, the concentrations of tumor necrosis factor-alpha (TNF-α), interleukin-1 beta (IL-1β), and interleukin-6 (IL-6) were determined using specific ELISA kits (Jining Industrial, Shanghai, China) following the manufacturer’s protocols [16].

### 2.7. Statistical Analysis

All measurements were conducted in triplicate, and the results are presented as mean ± standard deviation. Data were statistically analyzed using SPSS 23.0. A one-way analysis of variance (ANOVA) was employed to assess the statistical significance of the differences between groups. A *p*-value of less than 0.05 was considered significant. GraphPad Prism 9.0 and OriginPro 2023 were used for graphical plotting.

## 3. Results and Discussion

### 3.1. Extraction Yield and Chemical Composition

As shown in Table 1, different extraction methods significantly influenced the yield of polysaccharides. The extraction yields of DLPs ranged from 3.20% to 6.31%, which is notably higher than the yield reported by Yang et al., who found an extraction yield of 1.92% [30]. Enzyme-assisted extraction achieved the highest yield of 6.31 ± 0.39%, nearly double that of ADLP. This increased extraction yield can be attributed to the action of cellulase, which hydrolyzes and disrupts the cell wall structure, thereby enhancing the release of intracellular components [31]. Additionally, ultrasound-assisted extraction improved the yield by facilitating solvent penetration into the raw material through mechanical crushing and cavitation effects [32]. In contrast, alkali-assisted extraction resulted in the lowest yield, which was significantly lower than that of hot water extraction (*p* < 0.05). This phenomenon is attributed to the disruption of glycosidic bonds in the polysaccharides and the inhibition of the ethanol precipitation effect [33]. Furthermore, the composition analysis indicated the presence of proteins in these polysaccharide extracts. The polysaccharide content of UDLP was the highest, reaching 75.11%, followed by HDLP (Table 1). These data were consistent with the results of Li et al., who reported the carbohydrate content of *D. officinale* leaf polysaccharide was 77.04 ± 0.51% [34].

### 3.2. Molecular Weight

**Figure 2:**
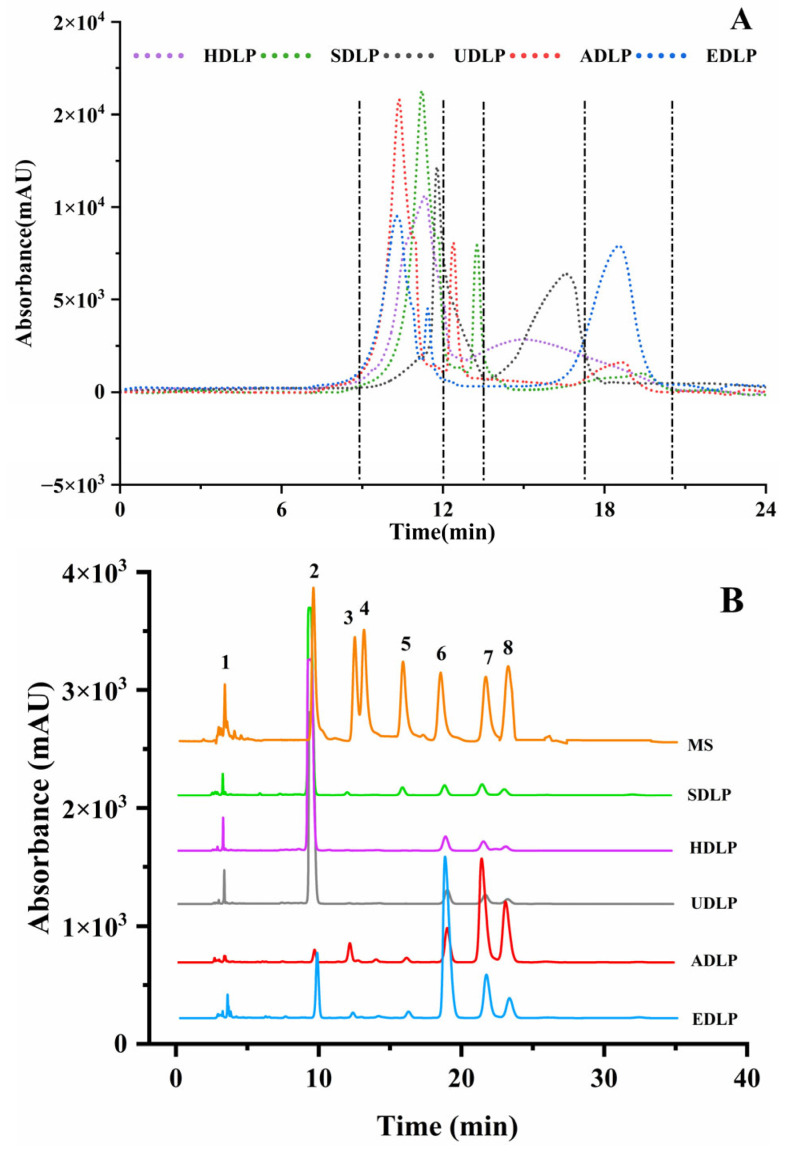

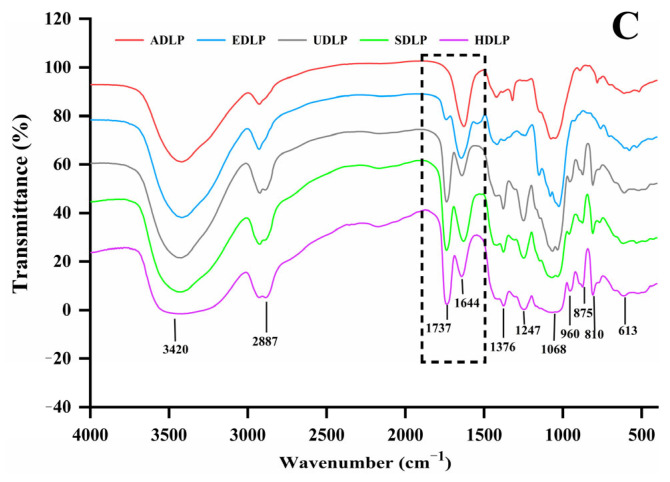
Spectra of molecular weight (**A**), monosaccharide composition ((**B**), MS indicates the standards; 1. PMP, 2. Man, 3. Rha, 4. GlcN, 5. GlcUA, 6. GalA, 7. Glc, 8. Gal) and FT-IR spectra (**C**) of polysaccharides from *Dendrobium officinale* leaves extracted using different methods.

The homogeneity of the relative molecular weight is a direct indicator of the purity of the polysaccharides, and its magnitude is closely associated with biological activity. Various extraction methods exert different impacts on the chain structure and glycosidic linkages of polysaccharides, thereby affecting their molecular weights. As illustrated in Figure 2A and detailed in Table 1, HDLP predominantly consisted of two components with molecular weights of 1.80 × 10^6^ Da (67.36%) and 9.02 × 10^4^ Da (32.64%), respectively. In contrast, the other four extraction methods resulted in varying degrees of reduction in the molecular weights of the polysaccharides. Notably, UDLP exhibited two major peaks of 6.56 × 10^5^ Da (89.41%) and 1.94 × 10^4^ Da (10.59%), which are significantly lower than those observed for HDLP. This indicates that ultrasound treatment can effectively reduce the molecular weight of polysaccharides by disrupting the chain structure and glycosidic bonds [35]. Furthermore, the HPLC analysis of ADLP, SDLP, and EDLP revealed three distinct peaks, suggesting the possible degradation of polysaccharide components during acidic, alkaline, and enzyme-assisted extraction processes. Nonspecific hydrolysis of acid/alkali and incomplete hydrolysis by enzymes could lead to the generation of additional polysaccharide components. This finding is consistent with the report on the extraction of corn silk polysaccharide through acid and alkali methods [36], as well as the study conducted by Zhao et al. on the enzyme-assisted extraction of polysaccharides from *Dioscorea opposita* Thunb. peel [18].

### 3.3. Monosaccharide Composition

The monosaccharide composition of DLPs obtained through the various extraction methods is summarized in Table 1. It is evident that different extraction methods yield varying predominant monosaccharide units and differing proportions (Figure 2B). Consistent with the previous research findings [5,30], mannose (83.72 ± 0.90%) was identified as the predominant monosaccharide in HDLP, followed by galacturonic acid (9.83 ± 0.40%). In comparison to HDLP, UDLP exhibited a nearly identical monosaccharide composition. This observation suggests that ultrasonic-assisted extraction does not significantly impact the type of monosaccharides present in DLPs but alters their proportions. Similar results have been reported in the comparison of hot water and ultrasound-assisted extraction for *Choerospondias axillaris* peel polysaccharides [37]. Additionally, a similar monosaccharide composition was also observed in SDLP; however, it exhibited a lower concentration of mannose and a higher concentration of glucuronic acid (Table 1). The increase in glucuronic acid may be attributed to the acid solution facilitating the hydrolysis of glycosidic bonds, while inhibiting the degradation of uronic acid [38]. In contrast, ADLP and EDLP demonstrated distinct monosaccharide compositions. Specifically, the major monosaccharides in ADLP were identified as glucose (41.93 ± 0.32%), galactose (28.94 ± 0.26%), and galacturonic acid (18.21 ± 0.03%), indicating that the alkali solution markedly hydrolyzes the polysaccharide chains and disrupts the hydrogen bonds between molecules [39]. Furthermore, galacturonic acid (71.37 ± 0.17%) was found to be the predominant monosaccharide of EDLP, followed by mannose and galactose. Enzymes have the potential to convert insoluble cellular carbohydrates into soluble forms, thereby altering the monosaccharide composition of the polysaccharides [18].

### 3.4. FT-IR

FT-IR is widely acknowledged as an effective technique for analyzing the functional groups in chemical structures. Figure 2C shows the FT-IR spectra of five distinct DLPs in the range of 4000~400 cm^−1^. The peaks at 3420 cm^−1^ and 2887 cm^−1^ are attributed to the stretching vibrations of hydroxyl (O-H) and carbon-hydrogen (C-H) bonds, respectively. Notably, no significant variations in these peaks were detected in the spectra of the five DLPs obtained through different extraction methods. The peak, around approximately 1737 cm^−1^, corresponds to the C=O vibration, which was prominently observed in HDLP, SDLP, and UDLP, but exhibited a weaker presence in EDLP. Conversely, this peak was absent in the spectrum of ADLP. Such discrepancies suggest that the extraction techniques employed may affect the stretching of esterified carboxylate groups. The absence of this peak implies that alkaline conditions likely induced a de-esterification effect on the DLPs [40]. The peak at 1644 cm^−1^ is indicative of the asymmetric bending vibration of a C=O group, suggesting the presence of uronic acids [41]. This peak can be observed in all five DLPs, indicating that uronic acid is present in all DLPs. Furthermore, the bands around 1247 cm^−1^ are characteristic of the symmetric C-O-S stretching vibrations, with a notable reduction in absorption observed in ADLP and EDLP. The pronounced bands between 1200 cm^−1^ and 1000 cm^−1^ result from the overlapping ring vibrations and the stretching vibrations of C-O-H side groups, as well as the C-O-C glycosidic bond vibrations, suggesting the potential presence of pyranose in the DLPs [16,42]. The distinctive absorption peak at 960 cm^−1^ confirms that β-type glycosidic bonding is the predominant bond type in DLPs, while the characteristic absorption peaks at 875 cm^−1^ and 810 cm^−1^ are attributed to the possible presence of D-glucopyranose and α-D-mannopyranose, respectively [16].

### 3.5. Congo Red Test

**Figure 3:**
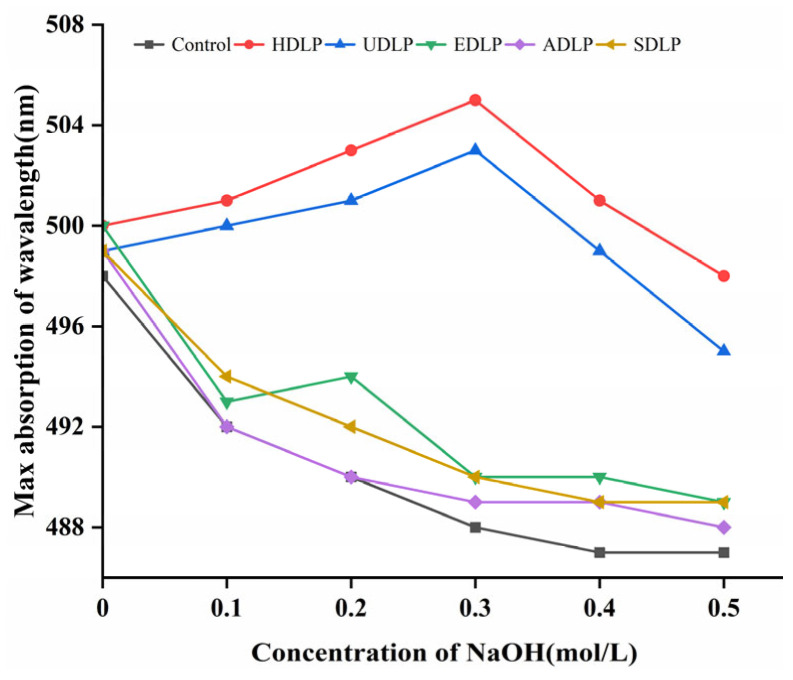
Congo red test results of polysaccharides from *Dendrobium officinale* leaves extracted using different methods.

The identification of the triple helical structure of polysaccharide is often achieved through the Congo red test, which facilitates the formation of a complex between Congo red and polysaccharides exhibiting a triple helix configuration. This interaction results in a red shift in the maximum absorption wavelength of the solution [43]. As shown in Figure 3, only HDLP and UDLP produced a significant red shift in the maximum absorption wavelength of the Congo red solution, whereas other DLPs did not demonstrate this phenomenon. This observation suggests that only HDLP and UDLP possess a triple helix conformation, consistent with the findings reported by Geng et al. [22]. The stability of the triple helix structure in polysaccharide solutions depends on the presence of intramolecular and intermolecular hydrogen bonds, which can be influenced by different extraction methods [43]. The results of this study indicate that acid-, alkali-, and cellulase-assisted extraction compromise the structural integrity of the triple helix configuration in DLPs.

### 3.6. SEM Analysis

**Figure 4:**
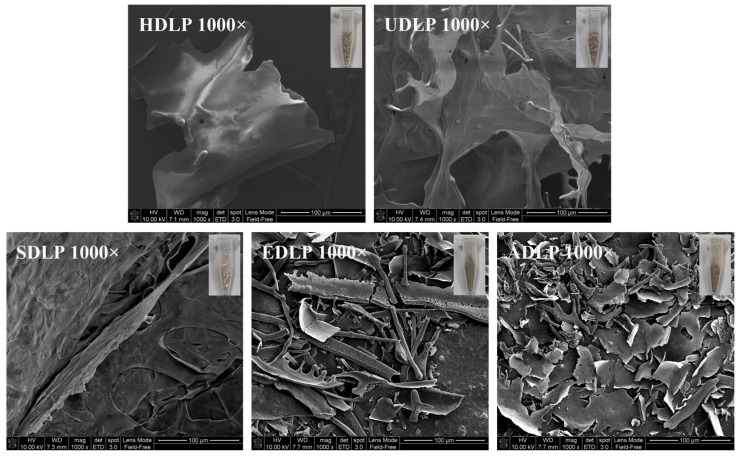
SEM images of polysaccharides from *Dendrobium officinale* leaves obtained by different extraction methods.

As reported by He et al. [12], the microscopic characteristics of *D. officinale* stem polysaccharides were influenced by the extraction methods employed. Our study corroborated these findings. As illustrated in Figure 4, HDLP exhibited an irregular block sheet-like formations with a smooth and flat surface. In contrast, the ultrasound waves resulted in the fragmentation of parts of the large sheet structures into small pieces, which were identified in UDLP. SDLP displayed a dense and multilayered microstructure with a rough texture, while both EDLP and ADLP exhibited loose fragments. EDLP presented irregular structures resembling rods, sticks, and strips, whereas ADLP displayed a more uniform, flaky structure. These observations regarding the microstructural characteristics can be attributed to the varying degrees of hydrolysis of the polysaccharide molecular chains caused by the distinct extraction methods employed [31].

### 3.7. In Vitro Adsorption Capacity

**Figure 5:**
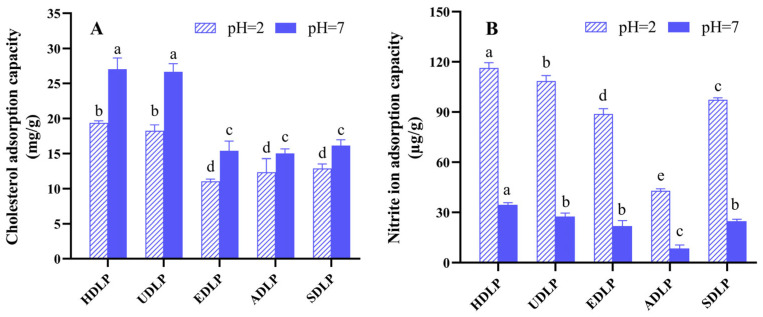
Adsorption capacities for cholesterol (**A**) and sodium nitrite (**B**) of polysaccharides from *Dendrobium officinale* leaves extracted using different methods. Different letters indicate the significance at *p* < 0.05. ANOVA and Duncan’s test were used to assess significant differences among the different groups.

The combination of bile acids and polysaccharides has been shown to enhance the excretion of bile acids and promote cholesterol metabolism, thereby contributing to a reduction in total cholesterol levels and the risk of cardiovascular diseases. As illustrated in Figure 5A, all five DLPs demonstrated superior adsorption capacity in a neutral environment (pH 7) compared to an acidic environment (pH 2). These results indicate that DLPs are more effective at adsorbing cholesterol in the intestinal tract. Furthermore, DLPs exhibited a significantly greater ability to absorb nitrite at pH 2 (Figure 5B), which contrasts with their adsorption behavior for cholesterol. This finding suggests that the stomach environment is more favorable for DLPs to adsorb NO^2−^, consistent with the previous reports [25]. Among all the samples tested, HDLP exhibited the highest nitrite adsorption capacity, followed by UDLP, SDLP, EDLP, and ADLP under both pH conditions. Notably, HDLP and UDLP demonstrated significantly higher adsorption effects for both cholesterol and nitrite compared to the other three DLPs under the test conditions (*p* < 0.05), which may be attributed to their structural characteristics.

### 3.8. In Vitro Antioxidant Capacity

**Figure 6:**
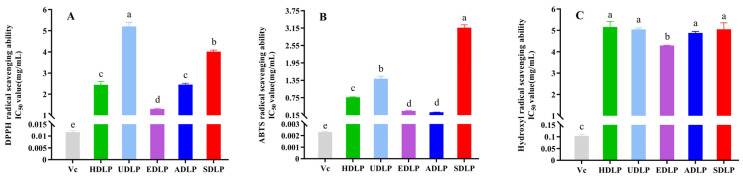
Antioxidant activities of polysaccharides from *Dendrobium officinale* leaves extracted using different methods. (**A**) DPPH radical scavenging ability; (**B**) ABTS radical scavenging ability; (**C**) Hydroxyl radical scavenging ability. Different letters indicate the significance at *p* < 0.05. ANOVA and Duncan’s test were used to assess significant differences among the different groups.

Numerous studies have shown that scavenging reactive oxygen species (ROS) is beneficial for the prevention of diabetes, cancer, and cardiovascular diseases. Plant polysaccharides are recognized as natural antioxidants [22], and previous research has demonstrated that water-extracted polysaccharides from *D. officinale* leaves possess excellent antioxidant properties [5]. However, different extraction methods may alter the antioxidant efficacy of these polysaccharides [31,36]. In the present study, we evaluated the antioxidant activities of DLPs extracted using different methods, focusing on their ability to scavenge DPPH, ABTS and hydroxyl radicals. The findings revealed that all five DLPs demonstrated antioxidant activities, with EDLP exhibiting the most effective ROS scavenging performance (Figure 6). The IC_50_ value for EDLP in scavenging DPPH radicals was significantly lower than that of any other DLPs (*p* < 0.05, Figure 6A). For ABTS radical scavenging, both EDLP and ADLP showed a significant advantage over the remaining samples (*p* < 0.05, Figure 6B), with IC_50_ values increasing in the order of HDLP < UDLP < SDLP. Additionally, EDLP displayed markedly superior scavenging activity towards hydroxyl radicals compared to the other samples (*p* < 0.05), although no significant differences were noted among the four DLPs regarding their hydroxyl radical scavenging capacities (Figure 6C). Therefore, EDLP demonstrated the most pronounced radical scavenging ability, indicating that cellulase-assisted extraction is advantageous for producing DLPs with robust antioxidant activity. The enhanced radical scavenging capacity of EDLP may be closely associated with its elevated galacturonic acid content, which is known to enhance the antioxidant activity of polysaccharides [19].

In contrast, no significant differences were observed among these four polysaccharides regarding their hydroxyl radical scavenging capacities (Figure 6C). For ABTS radical scavenging (Figure 6B), A-HDLP and E-HDLP demonstrated a significant advantage over the other three samples (*p* < 0.05), with the IC_50_ values significantly increasing in the order of HDLP < U-HDLP < S-HDLP. Summarily, E-HDLP exhibited the most prominent radical scavenging capacity, suggesting that cellulase-assisted extraction is beneficial for the preparation of *D. officinale* leaves polysaccharide with high antioxidant activity. The enhanced radicals scavenging capacity of E-HDLP may be attributed to its higher galacturonic acid content, which is known to enhance the antioxidant activity of polysaccharides [20].

### 3.9. Immunostimulatory Capacity

#### 3.9.1. Cell Proliferation Activity and Phagocytosis

**Figure 7:**
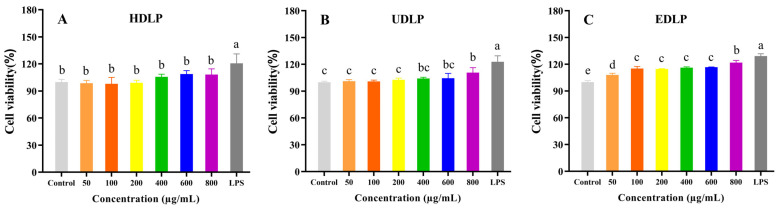

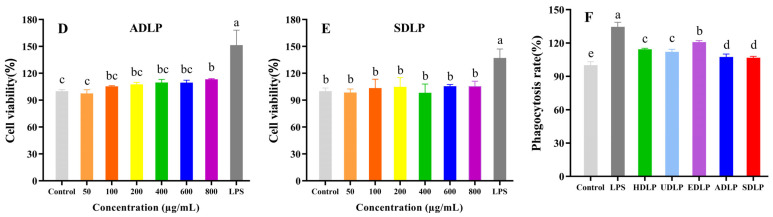
Effects of polysaccharides from *Dendrobium officinale* leaves extracted by different methods on cell viability (**A**), HDLP; (**B**), UDLP; (**C**), EDLP; (**D**), ADLP; (**E**), SDLP and cell phagocytosis (**F**) of RAW264.7 macrophages. Different letters indicated the significance at *p* < 0.05. ANOVA and Duncan’s test were used to assess significant differences among the different groups.

Macrophages play a crucial role in the immune response, and RAW264.7 macrophages are frequently utilized for in vitro studies of immune activity [44]. The effects of DLPs extracted by different methods on the proliferation of RAW264.7 cells were compared, with the results presented in Figure 7. Compared to the control group, cell viability in the DLP treatment groups increased to varying degrees at the tested concentrations (50–800 μg/mL), indicating that DLPs exhibit no cytotoxicity and have the potential to enhance the proliferation of RAW264.7 macrophages. However, the proliferation-enhancing effects of HDLP (Figure 7A) and SDLP (Figure 7E) were insignificant (*p* > 0.05) at the tested concentrations. In contrast, the proliferation-enhancing effects of EDLP were significant (*p* < 0.05) at all the tested concentrations (Figure 7C), while the effects of UDLP (Figure 7B) and ADLP (Figure 7D) were significant (*p* < 0.05) only at the high concentration of 800 μg/mL. Phagocytosis by macrophages is essential for non-specific immunity against the invasion of foreign materials [45]. Increased phagocytosis is a key indicator of macrophage activation and represents one of the primary defense mechanisms employed by these immune cells. As shown in Figure 7F, all five DLPs significantly increased the phagocytic activity of RAW264.7 cells (*p* < 0.05). Among these, EDLP demonstrated the strongest effect, boosting the phagocytosis of RAW264.7 cells by 20.79%, followed by HDLP (14.33%) and UDLP (12.10%).

#### 3.9.2. NO, TNF-α, IL-6, and IL-β Determination

**Figure 8:**
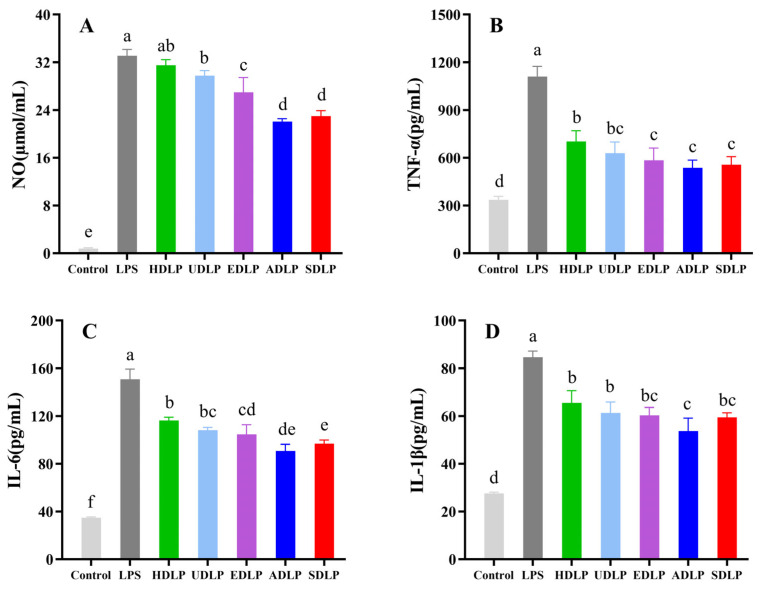
Effects of *Dendrobium officinale* leaf polysaccharides extracted using different methods on NO release (**A**), and the secretion of cytokines including TNF-α (**B**), IL 6 (**C**) and IL-1β (**D**) in RAW264.7 macrophages. Different letters indicated the significance at *p* < 0.05. ANOVA and Duncan’s test were used to assess significant differences among the different groups.

NO is an important gaseous signaling molecule that is integral to both physiological and pathological processes, particularly in inflammatory responses. TNF-α serves as a pivotal inflammatory mediator that plays a critical role in immune regulation by influencing the secretion of other inflammatory factors, activating T cells, and stimulating phagocytosis. IL-1β and IL-6 are potent pro-inflammatory cytokines that can stimulate the production of various other pro-inflammatory mediators [16]. Collectively, these factors are essential in modulating the immune response and enhancing the body’s defense against infections and injuries. Polysaccharides, as active substances, have the ability to stimulate macrophages to secrete NO, TNF-α, IL-6, and IL-1β, thereby bolstering the body’s resistance to external pathogens [46]. As demonstrated in Figure 8, all polysaccharide samples significantly promoted the release of NO and the secretion of TNF-α, IL-6, and IL-1β in RAW264.7 cells when compared to the control group (*p* < 0.05), although their effects were lower than those in the LPS treatment group. In terms of NO release, the stimulatory effects of HDLP, UDLP, and EDLP were significantly higher than those of SDLP and ADLP (*p* < 0.05), with the HDLP group achieving the highest level of 31.01 µmol/L, comparable to the LPS group (Figure 8A). For TNF-α (Figure 8B) and IL-6 (Figure 8C) secretion, the effects of HDLP and UDLP were significantly greater than those of EDLP, SDLP, and ADLP (*p* < 0.05), with no significant difference between UDLP and EDLP. Regarding IL-1β secretion, the effects of HDLP, UDLP, EDLP, and SDLP were significantly (*p* < 0.05) greater than that of ADLP (Figure 8D). Overall, the enhancing effects on NO release and the secretion of TNF-α, IL-6, and IL-1β in RAW264.7 cells were ranked in the following order: HDLP > UDLP > EDLP > SDLP > ADLP.

The immunomodulatory properties of polysaccharides are significantly influenced by their molecular structure, molecular weight, monosaccharide composition, and the types of glycosidic linkages present. Previous studies have demonstrated that the triple helix conformation of polysaccharides is crucial for the enhancement of immune responses [44]. Among the five DLPs, only HDLP and UDLP exhibited a triple helix structure, which may facilitate a more pronounced increase in cytokine secretion in RAW264.7 cells. Additionally, polysaccharides with a high molar ratio of mannose are more readily recognized by mannose receptors, complement receptor 3, or Toll-like 2 receptors, thereby exhibiting enhanced immunoregulatory capabilities [47]. The HDLP and UDLP molecules were found to possess a high mannose ratio (83.09% and 82.86%, respectively), which may further contribute to their superior immunomodulatory effects compared to the other polysaccharides. Moreover, molecular weight is another critical factor influencing the immunological activity of polysaccharides [48]. Polysaccharides with high molecular weight tend to stimulate increased levels of NO and cytokines in macrophages, as evidenced by studies on *Chlorella ellipsoidea* polysaccharide [47] and *Cordyceps militaris* polysaccharide [49]. In comparison to UDLP, HDLP exhibited a higher molecular weight, which was associated with its enhanced immunostimulatory activity.

## 4. Conclusions

In summary, extraction methods significantly influenced the physicochemical properties of DLPs, as well as their bioactivities. Both HDLP and UDLP retained a triple helix structure, demonstrating strong adsorption capabilities for cholesterol and nitrite, marking them as promising candidates for the prevention of hyperlipidemic diseases. Additionally, HDLP and UDLP exhibited higher immunostimulatory activity, as evidenced by their ability to enhance the release of NO and the secretion of cytokines in macrophages. Nevertheless, EDLP contained the highest content of galacturonic acid and displayed superior scavenging capacities towards free radicals. These findings suggest that the selection of an extraction method for DLP should be tailored to meet the specific requirements of various practical applications.

## Figures and Tables

**Table 1:** The yield, chemical composition, monosaccharide composition, and molecular weight distributions of polysaccharides from *Dendrobium officinale* leaves extracted using different extraction methods.

	HDLP	UDLP	SDLP	ADLP	EDLP
Extraction yield (%)	4.76 ± 0.14 ^c^	5.22 ± 0.19 ^bc^	5.40 ± 0.20 ^b^	3.20 ± 0.23 ^d^	6.31 ± 0.39 ^a^
Chemical composition (%, *w*/*w*)					
Polysaccharide	74.20 ± 4.45 ^a^	75.11 ± 0.99 ^a^	69.36 ± 1.20 ^b^	55.37 ± 1.57 ^c^	52.10 ± 2.75 ^c^
Protein	9.17 ± 0.14 ^b^	9.48 ± 0.45 ^b^	2.65 ± 0.10 ^d^	8.56 ± 0.42 ^c^	11.02 ± 0.51 ^a^
Monosaccharide composition (%, mol)					
Mannose	83.72 ± 0.90 ^a^	83.31 ± 0.89 ^a^	75.78 ± 1.22 ^b^	2.08 ± 0.02 ^d^	7.47 ± 0.03 ^c^
Rhamnose	0.23 ± 0.01 ^d^	0.25 ± 0.04 ^d^	1.45 ± 0.08 ^b^	6.48 ± 0.19 ^a^	1.24 ± 0.05 ^c^
Glucosamine	0.53 ± 0.01 ^b^	0.39 ± 0.24 ^b^	0.44 ± 0.02 ^b^	0.88 ± 0.19 ^a^	0.59 ± 0.06 ^b^
Glucuronic acid	0.24 ± 0.00 ^c^	0.23 ± 0.00 ^c^	3.56 ± 0.16 ^a^	1.49 ± 0.09 ^b^	1.50 ± 0.08 ^b^
Galacturonic acid	9.83 ± 0.40 ^c^	10.07 ± 0.04 ^c^	10.02 ± 0.67 ^c^	18.21 ± 0.03 ^b^	71.37 ± 0.17 ^a^
Glucose	1.28 ± 0.46 ^d^	1.32 ± 0.45 ^d^	3.09 ± 0.76 ^c^	41.93 ± 0.32 ^a^	10.84 ± 0.14 ^b^
Galactose	4.16 ± 0.75 ^d^	4.19 ± 0.30 ^d^	5.66 ± 0.03 ^b^	28.94 ± 0.26 ^a^	7.00 ± 0.02 ^c^
Molecular weight (Da)					
Peak (Ι)	1.80 ± 0.15 × 10^6^	6.56 ± 0.13 × 10^5^	1.47 ± 0.03 × 10^6^	2.74 ± 0.14 × 10^6^	2.55 ± 0.02 × 10^6^
Area (%)	67.37 ± 3.14	10.59 ± 0.96	64.29 ± 2.60	69.91 ± 3.55	41.65 ± 0.27
Peak (II)	9.02 ± 0.67 × 10^4^	1.94 ± 0.14 × 10^4^	9.85 ± 0.76 × 10^4^	5.01 ± 0.22 × 10^5^	1.04 ± 0.02 × 10^6^
Area (%)	32.63 ± 3.14	89.41 ± 0.96	34.94 ± 1.38	12.75 ± 0.50	5.77 ± 0.08
Peak (III)	ND	ND	0.48 ± 0.16 × 10^3^	4.1 ± 0.29 × 10^3^	3.31 ± 0.03 × 10^3^
Area (%)	ND	ND	0.39 ± 0.01	17.35 ± 4.05	52.58 ± 0.32

HDLP, hot water extracted *D. officinale* leaf polysaccharide (DLP); UDLP, ultrasound-assisted extracted DLP; SDLP, acid extracted DLP; ADLP, alkali extracted DLP; EDLP, enzyme-assisted extracted DLP. Different letters in each row indicate statistically significant differences (*p* < 0.05). Analysis of variance (ANOVA) and Duncan’s test were used to assess significant differences among the different extraction methods. The abbreviation of ND denotes “not detected”.

## Data Availability

The original contributions presented in this study are included in the article. Further inquiries can be directed to the corresponding authors.
